# A Wireless Underground Sensor Network Field Pilot for Agriculture and Ecology: Soil Moisture Mapping Using Signal Attenuation

**DOI:** 10.3390/s22103913

**Published:** 2022-05-21

**Authors:** Srinivasa Balivada, Gregory Grant, Xufeng Zhang, Monisha Ghosh, Supratik Guha, Roser Matamala

**Affiliations:** 1 Pritzker School of Molecular Engineering, University of Chicago, Chicago, IL 60637, USA; balivadas@uchicago.edu (S.B.); gdgrant@uchicago.edu (G.G.); mghosh3@nd.edu (M.G.); guha@uchicago.edu (S.G.); 2 Materials Science Division, Argonne National Laboratory, Lemont, IL 60439, USA; xu.zhang@northeastern.edu; 3 Center for Nanoscale Materials, Argonne National Laboratory, Lemont, IL 60439, USA; 4 The Consortium for Advanced Science and Engineering, University of Chicago, Chicago, IL 60637, USA; 5 Department of Electrical and Computer Engineering, Northeastern University, Boston, MA 02115, USA; 6 Department of Electrical Engineering, University of Notre Dame, Notre Dame, IN 46556, USA; 7 Environmental Science Division, Argonne National Laboratory, Lemont, IL 60439, USA

**Keywords:** deep learning, RSSI, radio frequency attenuation, wireless underground sensor network system

## Abstract

Wireless Underground Sensor Networks (WUSNs) that collect geospatial in situ sensor data are a backbone of internet-of-things (IoT) applications for agriculture and terrestrial ecology. In this paper, we first show how WUSNs can operate reliably under field conditions year-round and at the same time be used for determining and mapping soil conditions from the buried sensor nodes. We demonstrate the design and deployment of a 23-node WUSN installed at an agricultural field site that covers an area with a 530 m radius. The WUSN has continuously operated since September 2019, enabling real-time monitoring of soil volumetric water content (VWC), soil temperature (ST), and soil electrical conductivity. Secondly, we present data collected over a nine-month period across three seasons. We evaluate the performance of a deep learning algorithm in predicting soil VWC using various combinations of the received signal strength (RSSI) from each buried wireless node, above-ground pathloss, the distance between wireless node and receive antenna (D), ST, air temperature (AT), relative humidity (RH), and precipitation as input parameters to the model. The AT, RH, and precipitation were obtained from a nearby weather station. We find that a model with RSSI, D, AT, ST, and RH as inputs was able to predict soil VWC with an R^2^ of 0.82 for test datasets, with a Root Mean Square Error of ±0.012 (m^3^/m^3^). Hence, a combination of deep learning and other easily available soil and climatic parameters can be a viable candidate for replacing expensive soil VWC sensors in WUSNs.

## 1. Introduction

Wireless Underground Sensor Networks (WUSNs) have been increasingly studied over the past two decades for terrestrial, agricultural, and ecological applications [1,2,3,4,5,6,7,8,9], including the demonstration of a fully buried, spatially distributed sensor node network that will not disrupt agricultural and/or industrial processes [10,11,12,13,14,15,16,17]. Such sensor networks have been explored as the backbone for geospatial internet-of-things (IoT) for agricultural applications [18]. Data gathered from such networks, combined with data curation and artificial intelligence-based analysis, is anticipated to be a significant component of future digital farming and ecological practices provided challenges in cost and scalability are met [18,19,20,21,22]. There are two important areas of WUSN applicability in agriculture and land ecology that require further examination. First, there is the need to test WUSN performance under field conditions over an extended period to examine robustness and scalability. The second area relates to the potential use of the electromagnetic wireless signal, currently only used for data transmission, to become an indicator of the soil environmental conditions, as the signal strength has been shown to be sensitive to variations in soil water content and other soil factors [10,11].

Soil volumetric water content (VWC) is a crucial parameter of interest for many applications in ecology, soil science, and agriculture [23,24]. Methods available for capturing the soil–hydrological system’s natural heterogeneity are unsatisfactory at the 1 to 1000 m^2^ scales. For instance, remote sensing measurements are limited to low spatial and temporal resolutions [25], while point measurement sensors sample small volumes, are relatively expensive (typically a few hundred US dollars each), and individually do not reflect the heterogeneity of the soil. Thus, there is a need for soil VWC sensor systems that can operate in this intermediate spatial scale at high temporal resolutions.

The primary goal of our work reported in this paper is to show that a WUSN-based IoT system with soil data sensing, curation, and mapping can be successfully and continuously operated in farms under “real-life” conditions. We demonstrate this by presenting results from a nine-months pilot experiment in which we have designed and operated a 23-sensor WUSN over a 130-acre site in an active soy/corn farm. An additional goal of our work has been to show that using a combination of RSSI data and machine learning makes it possible to map the soil VWC without the need for in-ground point moisture sensors, which can be cost prohibitive for large networks.

### 1.1. Related Work

In WUSNs, the communication hardware within the buried sensor nodes communicates with a base station through a combination of transmission through the soil and the atmosphere. Akyildiz and Stuntebeck have described the opportunities and challenges in WUSN technology [2]. Different types of topologies (e.g., underground-to-underground and underground-to-aboveground) have been modeled and tested experimentally [2,5,15,26,27], and the effect of soil properties on wireless underground signal propagation is well-studied [5,28,29,30,31,32]. Studies have included examining power consumption [30,33,34,35], as well as the deployment of communication protocols such as Mica [14], Zigbee [36], Wi-Fi [37,38], RFID [39], cellular [37], Sigfox [10], and Lora [12,35] for WUSNs. While experimental WUSN evaluations have been carried out in laboratory testbeds [9,22,40,41,42] and in some outdoor environments [10,12,43,44,45,46] and agricultural farms [14,19,32,43,47,48,49,50], testing has been limited, often with one or a few sensor nodes. There are no studies of WUSNs deployed deep in the vadose zone and tested under “real-life” conditions, long time periods, including multiple seasons, and over large areas of active agricultural management.

Soil moisture is an important input for flood and drought forecasting, in soil ecological studies, and in precision agriculture for effective nutrient management [51,52,53,54,55,56,57]. For this purpose—as noted earlier—it is important to be able to measure soil moisture at high resolutions (1–1000 m^2^), and efficient techniques for this are lacking.

Different machine learning-based models have been developed with varying degrees of success for soil moisture estimation using a variety of environmental inputs (such as air and soil temperature, relative humidity, etc.), with some requiring initial soil moisture measurements as inputs [25,58,59,60,61,62,63,64,65,66]. In addition, it has been pointed out that the Relative Signal Strength Indicator (RSSI) parameter, which quantifies signal attenuation through soil, is an easily measurable variable for any WUSN and can be a powerful input for soil moisture measurement. Microwave propagation through soil is strongly affected by the soil’s dielectric properties [67], which in turn is affected by the soil texture, and the amount of organic matter and water present in the soil. This latter effect can be strong, since water and soil have significantly different dielectric constants of ~80 and 3–5, respectively. Small changes in soil moisture can therefore affect the propagation of the WUSN signal [68] and can be detected via changes in RSSI.

Several researchers have studied the relationship between attenuation of the electromagnetic signal and soil moisture [10,11,36,38,69]. Soil moisture determination via RSSI changes would enable high resolution measurements without relying upon expensive soil sensors. Aroca et al. [70] used a buried passive RFID network and calibrated a neural network model for soil moisture prediction using RSSI as an input. However, due to a limited transmission range, the technique needs proximal signal reception via a mobile robot and can therefore be limited in scalability and resolution. Rodic et al. [33] installed a low-power, LoRa-based, underground sensor node and two above-ground gateways, and optimized deep learning techniques to predict soil moisture from the RSSI at one buried location. All these experiments were carried out under limited and controlled conditions or did not explore the mapping of moisture using RSSI across large areas enabled by multiple-sensor WUSNs. Since signal attenuation can also depend upon the field’s topology, seasonal conditions, various other field conditions (such as crop coverage), and the system’s physical parameters [1,10,17]), the relationship between RSSI and VWC requires field validation under realistic operating conditions—such work has been lacking so far.

### 1.2. Contribution of This Work

In the present study we offer two contributions pursuant to our research goals noted in the introduction section. Firstly, we demonstrate the design and deployment of a WUSN and then examine the results of 9 months of continuous operation of a fully buried, non-intrusive WUSN at an agricultural field located at Fermi National Laboratory (Fermilab, Batavia, IL, USA). Through this demonstration, we show that a WUSN can operate under all-season, realistic field conditions. The wireless network is based on the increasingly used unlicensed ISM band, deployed around 900 MHz (in the USA) and made popular through low-power IoT wireless networks such as LoRa and Sigfox. The WUSN collects and maps soil VWC, electrical conductivity (EC), and soil temperature (ST) simultaneously, and the data can be visualized in near real-time through a web-based user interface. We discuss the performance of the WUSN through seasonal weather variations (fall, winter, and spring) and the impacts of climate, soil, and site characteristics affecting the sensor module transmissions.

Secondly, we ask the question: Can attenuation of the wireless signal in-ground be used as a robust predictor of soil VWC under real-life conditions in a field and over changing seasonal climatic conditions? In doing so, we examine whether the RSSI, which is used to transmit the sensor measurements to the base antenna, could also be used to predict and spatially map soil VWC under long-term field testing. We found that, while RSSI alone was not able to predict soil VWC accurately over time, the addition of site-specific physical, soil, and climatic factors as input parameters along with the use of available, off-the-self machine learning algorithms significantly improves predictive performance for VWC. We evaluate three well-known machine learning techniques: support vector regression (SVR) [59,60], extreme learning machine (ELM) [61], and artificial neural network–multilayer perceptron (ANN–MLP) [59,66]. Among the tested models, ANN–MLP greatly increased the predictive capability for VWC. We propose this machine learning-based approach as an alternative method for estimating soil VWC with WUSNs, thereby obviating the need for expensive point sensors for VWC measurement (which can limit geographical scalability). We note that the scope of the paper is limited to exploring the efficacy of readily available machine learning algorithms in extracting soil moisture from our sensor network data, and not to carry out detailed research on the algorithms themselves.

The rest of the paper is organized as follows. Section 2 explains the sensor node development, WUSN architecture, and the experimental setup used in the work. Section 3 shows the preliminary results from the WUSN, wireless transmission, and effects of soil moisture and elevation and describes the performance results of soil moisture prediction using different machine learning techniques. The paper’s conclusion is in Section 4.

## 2. Materials and Methods

### 2.1. Sensor Node Development

**Figure 1:**
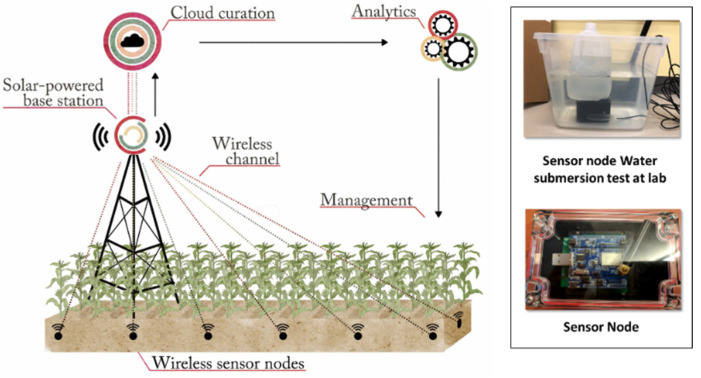
The wireless underground sensor network Thoreau 2.0 design. Inset shows sensor node and water submersion test conducted in the laboratory prior to field deployment.

Thoreau 2.0 is a modified version of an earlier system developed and operated at the University of Chicago campus described in Zhang et al. (2017) [11]. The wireless backbone of the Thoreau 2.0 system is a Sigfox 901.2 MHz low-power IoT wireless network, and the architecture contains three components: (1) the buried sensor nodes, (2) a base station, and (3) a user interface (Figure 1).

Each sensor node consists of a sealed carbonate casing (16 cm × 8 cm × 8.5 cm) that contains the electronics, power source, and a transmitting antenna (Figure 1 inset shows the electronic components and antenna, while the power rack is housed beneath the electronics and cannot be seen in the picture). Each node is connected to an external sensor that simultaneously measures soil VWC, ST, and EC (TEROS12, Meter Environment, Pullman, Washington, DC, USA) in the vicinity of the box. The mechanical design of the sensor node box is critical. Building upon our experience from the first-generation WUSN (Thoreau 1.0, [11]), a hermetically sealed box was designed (Windy City Lab, Chicago, IL, USA) that could withstand extreme water and temperature fluctuations. We used O-rings around the lid to prevent leaks and silicone sealant to waterproof the encasing. A watertight, nylon cable gland was used for cable connection to the external sensor. Additionally, a conformal coating was applied to all electronics inside the box. A humidity sensor inside the box transmits the sensor node internal humidity status of the box. The nodes have been running for more than 29 months (6 September 2019, to the time of paper submission) and have withstood high heat and cold conditions in the field.

The sensor node electronics consist of a micro controller unit (MCU) (Xenon, MCU V001) that manages the sensor node core functions, such as data acquisition, radio frequency (RF) transmission, and power use; and an RF transceiver (Sigfox Thinxtra board) that is integrated with an RF trace antenna and a power amplifier (transmission power ~22 dBm). Each sensor node is powered by a battery rack containing 4 AA lithium batteries. Power usage analysis indicates that each sensor node can be active for up to 4.5 years with one set of batteries.

**Figure 2:**
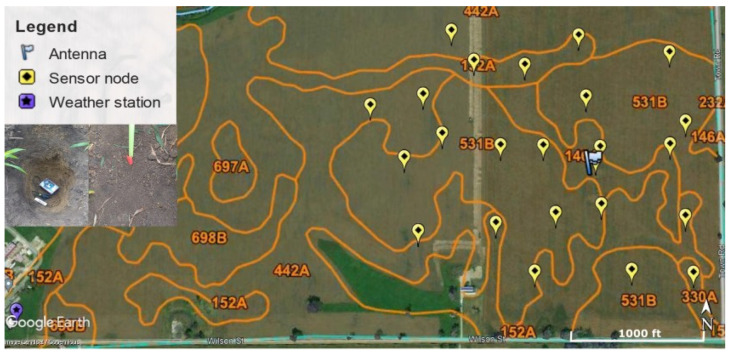
Sensor node locations (yellow pins), base station location (flag), weather station (purple pin), and soil types (orange lines and numbers denote USDA soil type assessment). Inserts show a sensor node deployed in the soil.

In the sensor nodes, as a way to conserve power, a watchdog timer awakens the MCU from sleep mode every 30 min. The MCU subsequently wakes up the sensor, collects a measurement, and encodes the data prior to transmitting to the base station through the RF transceiver. The RF transceiver initiates a transmission by sending three uplink packages in sequence on three random carrier frequencies to the base station. However, in order to conserve power, and based upon our experience with Thoreau 1.0, we modified the firmware to produce only two uplink packet transmissions for this project. A single, base station (Sigfox Macro, SBS-T-902v3) is installed in the center of the field, with an antenna mounted at a height of 10 m from the ground. The base station receives transmissions from nodes located anywhere in an approximately 530 m radius from the antenna. The base station is powered via a photovoltaic array with battery backup. The location of the base station antenna and the sensors are shown in Figure 2. The Sigfox IoT Network receives the signal from the base station. RSSI is then calculated by SigFox based on the average radio signal intensity of the packets correctly received per transmission. The RSSI varies over time as soil parameters change, and it is these natural variations that our machine learning algorithms identify during training to determine soil moisture from RSSI values.

The base station antenna receives the data packets from the sensor nodes and transmits them (via the internet) to a cloud-based backend operated by Sigfox. The data is then accessed (using a callback API, curated, and visualized on Thoreau (Thoreau-Home (uchicago.edu)) in near real-time. Thoreau is a cloud-based, open web interface [71] where all data is easily accessible and available for download by the public.

### 2.2. Experimental Setup

Thoreau 2.0 WUSN was installed in an agricultural field located at Fermilab. The field is cultivated utilizing corn–soybean crop rotation management, and the soil is tilled to a ~25 cm depth every other year after the corn is harvested. Twenty-three sensor nodes were deployed within a ~530 m radius area from the base station and were buried ~40 cm deep. The sensors deployed with each node were also buried at a ~40 cm depth, coplanar with the tops of the sensor node boxes. This depth was chosen to avoid interference with farming activities and to avoid sensor displacement and damage due to plowing (plowing depth is typically ~25 cm). Being a noninvasive system is an important consideration and requirement for many buried agricultural network applications. Soil motion can also occur due to “frost heave”, which occurs when ice lenses form in the soil, usually in cold environments with fine-textured soils. As the ice lenses grow, the soil can move upward due to pressure. In our study, the majority of the soils were of the Markham series. The sensors were buried 40 cm deep in the “B horizon”, which is classified as silty clay loam soil with 35–45% clay content and moderately textured. The amount of clay in our soils leads to lower hydraulic conductivity and small void volume. The resultant drop in capillary flow with clay content tends to reduce the severity of frost heave because it impedes ice lens formation [72]. Because of the moderate clay content in our soils, as well as the moderate texture, we do not expect significant frost heave over the time period of our experiments (~9 months). In addition, the antennas contained within the rugged sensor boxes are omnidirectional dipole antennas, and small motions in the soil cannot alter the RSSI readings significantly.

As mentioned in the previous section, each sensor node is integrated with sensors that simultaneously measure soil VWC, ST, and EC. Additionally, weather parameters, including air temperature (AT), relative humidity (RH), and precipitation (P) were obtained from a weather station located at Fermilab near the field site that collected measurements every 5 min (https://wwwesh.fnal.gov/pls/default/weather.html (accessed on 25 June 2020)) (Figure 2). We determined the soil type adjacent to each of the sensor nodes by using the Natural Resources Conservation Services Online Soil Survey (https://www.nrcs.usda.gov/wps/portal/nrcs/main/soils/survey/ (accessed on 15 December 2019)) to map the soil types within the field area. We superimposed this map and the sensors’ geographical coordinates onto a Google Earth (https://www.google.com/earth/ (accessed on 10 January 2020)) image of the field site area (Figure 2). Sensor nodes were adjacent to five soil types, Elliott (146A), Drummer (152A), Peotone (330A), Mundelein (442A), and Markham (531B) (Figure 2).

## 3. Results

### 3.1. Temporal Variation of Soil Properties

**Figure 3:**
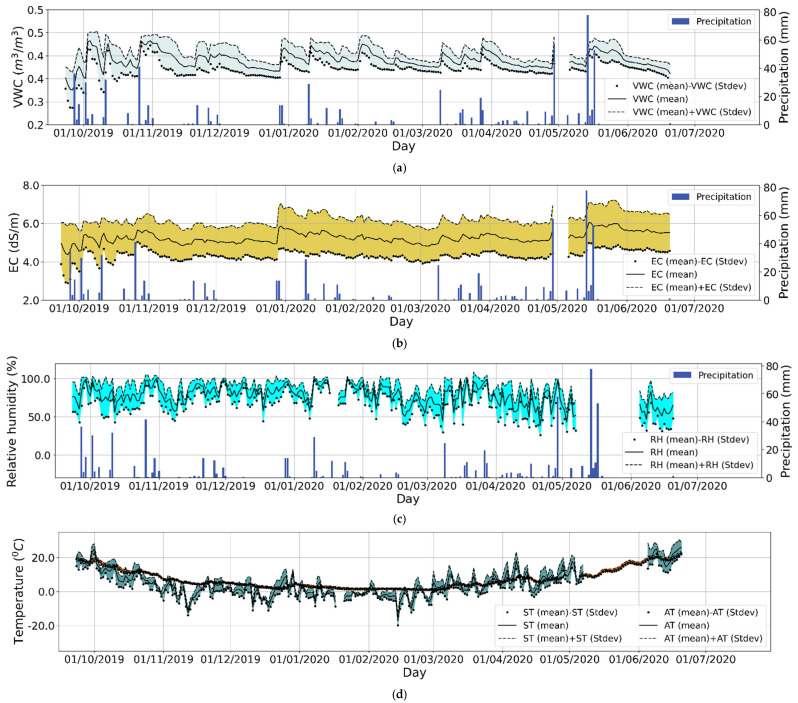
Daily mean averages ± standard deviation of soil volumetric water content (VWC) (**a**), electrical conductivity (EC) (**b**), relative humidity (**c**), and soil and air temperature (**d**). Relative humidity and air temperature measurements were collected from the weather station shown in Figure 2. Blue bars are cumulative daily precipitation events. Shadows indicates the ± standard deviation of the mean for VWC, EC, and RH.

Measurements were collected over 289 days from 6 September 2019 through 20 June 2020. During this time, the WUSN operated continuously and produced 112,000 measurements after data curation and QA/QC. Descriptive statistics for soil VWC, EC, and ST, and weather variables AT, RH, and P averaged for the three seasons comprising the study time are shown in Table 1. Figure 3 shows daily means for these variables and precipitation events.

The overall high average soil EC values of the field site indicate good soil fertility. The site is composed of silt loam and silty clay loam soils that are prime farmland soils. Nevertheless, the soils have a tendency of being too wet, potentially creating nutrient build up, as indicated by high maximum soil EC and VWC, particularly in the spring, as shown in Table 1. As expected, soil VWC increases after each precipitation event (Figure 3a). A positive correlation of soil VWC with EC was observed throughout the study time (R = 0.47; Pearson correlation analysis, MATLAB R2020b). Analyzing the data by season, the correlation coefficients of soil VWC with EC are 0.54, 0.45, and 0.29 in fall, spring, and winter, respectively (Pearson correlation analysis, MATLAB R2020b). This correlation is not surprising, as it is known that the more water there is in the soil the more cations are in solution and the soils’ capacity to conduct electricity increases, resulting in higher EC values. Weather variations in RH, ST, and AT occurred throughout the study time, with cold winter and warmer spring temperatures, and frequent fluctuations in air temperature and relative humidity typical of continental climates (Figure 3c,d).

### 3.2. System Performance

**Figure 4:**
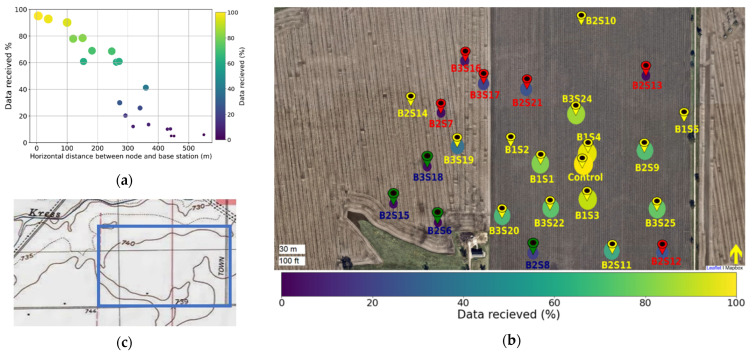
The average percentage of data packets received by the base station from the sensor nodes as a function of the horizontal distance from the base station (**a**) and from each sensor as they were located in the field (**b**). The size and color of the filled circles represents the percentage of data packets received. The flag indicates the base station location; yellow, red, and green pins represent sensors installed at 740 and 735 feet elevation and those installed in the flood plain zone of the study area, respectively (**b**). The soil topographic map (**c**) used to determine site elevation is retrieved from: https://www.usgs.gov/search-map?search=3DEP (accessed on 25 November 2020).

Electromagnetic wave transmissions can be attenuated by several factors, including distance between a sensor node and the base station [2,10]. Figure 4a,b show the percentage of data packets received by the base station from the sensors during the 9 months of the study. Results indicate that there is a substantial loss of data packets from the sensor nodes that are farthest away from the base station (Figure 4a). In addition, we found that the amount of data received is not equal across sensors installed the same distance from the base antenna. Analysis of the data packets received from each sensor superimposed onto a topographic map (Figure 4b,c) suggests that the percentage of data packets received is a function of sensor node distance to the base station, terrain elevation, and the localized concentration of soil moisture. For instance, sensors B3S20, B2S8, B2S11, B2S12, B2S13, and B2S21; and B3S19, B3S17, and B2S6 are installed approximately equidistant to the base station, but they differ in the number of data packets received by the base station. Sensor B2S8 (green pins in Figure 4b) is in a floodplain, leading to fewer data packets received compared to the nearest sensors B2S11 and B3S20, while sensors B2S7, B2S12, and B2S13 (red pins in Figure 4b) only differ in their topographic position and elevation yet have different data packet percentages (Figure 4b).

### 3.3. Spatial Variation of Volumetric Water Content Before, During, and after a Precipitation Event

**Figure 5:**
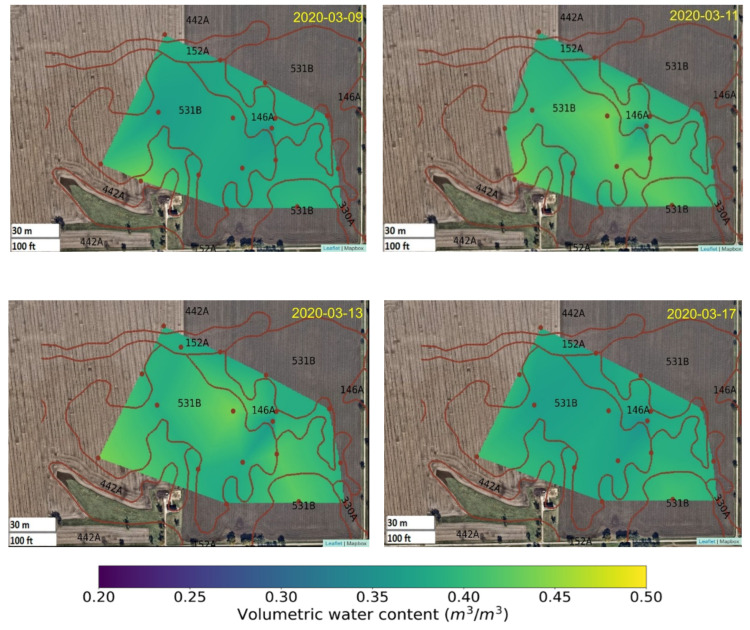
Volumetric water content (m^3^/m^3^) daily average intensity maps before and after 24.6 mm precipitation. Brown dots indicate sensor location and brown lines/numbers indicate soil types (brown lines and numbers denote USDA soil type assessment). Intensity maps were generated by linear interpolation between measured points.

Soil chemistry attributes vary from site to site due to the soil’s heterogenic nature, and are influenced by the water-holding capacity of soils and water availability. For instance, some soils dry faster than others because of their high porosity. Soils in northern Illinois are generally high in silt and clay, somewhat poorly drained, and have high soil water holding capacity because of high organic matter content. Humid climate conditions prevail in this area, which receives an average of 900 mm of rainfall per year, plus a variable amount of snowmelt in winter and spring. For farmers in this area, one of the most important needs is to know when the soil conditions are adequate for planting, since spring planting in overly wet conditions can result in surface compaction, leading to, in turn, poor plant emergence and root development (and reduced yield). WUSNs have the capability to determine the spatial distribution of soil moisture at any given time. For example, heatmaps on Figure 5 show the spatial distribution of soil VWC daily mean before and after a rainfall event. The heatmaps were generated by linearly interpolating between measured datapoints, as in Harris et al. (2020) [73], and overlaying the results over a base map obtained from the OpenStreet map API from MapBox [74]. Rain began on 9 March 2020, resulting in a 24.6 mm precipitation event. Although before it rained the soil VWC was already high, this event elevated soil VWC content from 0.36 to 0.41 m^3^/m^3^ ± 0.03 sensor accuracy for the next 3–4 days, after which soil VWC levels dropped back to 0.37 m^3^/m^3^ ± in situ sensor accuracy. Interpolation of measurements and mapping of soil conditions and attributes in almost near real-time is important and can inform agricultural management decisions, one of the many new applications for WUSN technologies.

### 3.4. Estimation of VWC from Received Signal Strength Indicator (RSSI)

**Figure 6:**
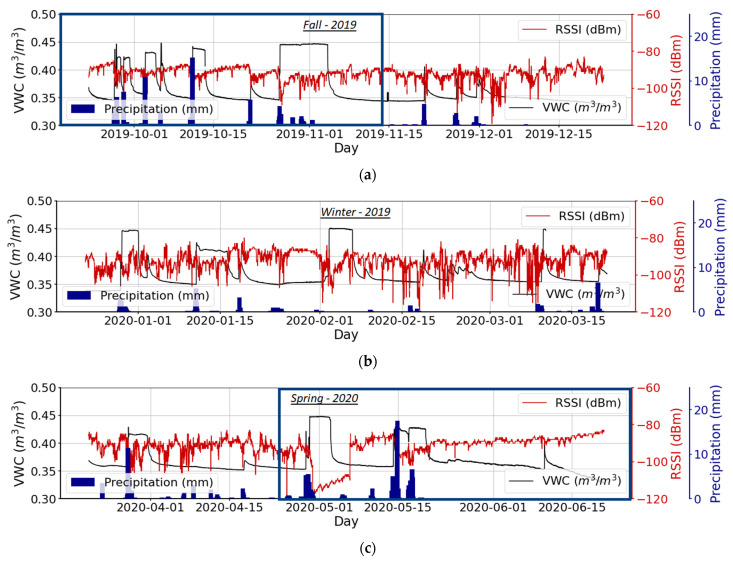
Measured RSSI (red line) and VWC (black line) by a sensor node buried 40 cm below the ground and located at 5 m to the base station during fall 2019 (**a**), winter 2019 (**b**), and spring 2020 (**c**) (from 6 September 2019 to 20 June 2020). Blue bars represent cumulative daily precipitation.

The RSSI value received by the base station is generally lower than the original sensor node transmission. This signal attenuation is the result of the electromagnetic wave traveling through the soil medium and the free-space path in the air and intervening vegetation. Variations in soil moisture also affect RSSI values because the presence of moisture alters the soil’s dielectric response to the propagation of the signal [10]. Figure 6 denotes RSSI values typically received by the base station, plotted against the soil VWC and the amount of precipitation at that time. As Figure 6 indicates, soil VWC rises following precipitation, sometimes reaching the upper detection limit of the sensor, and then decreases within a few hours to days due to soil water drainage and evaporation (when the soil is bare) or evapotranspiration (when plants are present). Correlation of soil VWC with RSSI is not present in winter, but it appears over the course of spring and early fall (Figure 6).

A correlation analysis using all data indicates a weak significant negative correlation (R = −0.25, *p*-value = <0.005) of soil VWC with RSSI. This analysis was conducted with a total of 92,000 datapoints, obtained after removal of soil VWC values that were above the maximum detection limit of the sensors, 0.45 m^3^/m^3^ (removal of 4000 datapoints), and of incomplete datasets due to temporary malfunction of the weather station that led to the removal of another 12,000 datapoints from our dataset. Because electromagnetic signal propagation is also impacted by other factors, such as terrain elevation and distance of the sensor node to the base station, as shown in Figure 4, and other factors identified in the literature, including climatic and weather factors [75,76], we investigated whether a nonlinear approach using standard machine learning algorithms could improve the prediction of soil VWC by taking into consideration RSSI and the various factors that might affect signal transmission from the sensors.

As noted earlier, we used an Artificial Neural Network with Multilayer Perceptron (ANN–MLP) algorithm (details in [58,77,78,79,80]) for constructing soil VWC predictive models. MATLAB R2020b was used for the neural network development, training, and simulations [81]. The total dataset was randomly classified into 70% for training (64,000 datapoints), 15% for validation (13,500 datapoints), and 15% for testing (13,500 datapoints). Soil VWC in situ measurements (collected by the external soil sensor) were used as ground truth data and target parameters for the model. Available input parameters that could be used to train the model included WUSN site parameters (RSSI and hypotenuse distance (D) between the buried sensor node and base station antenna) and soil (ST) and weather parameters (P, AT, and RH). Weather parameters were averaged at 30 min intervals to harmonize all data to that time interval for the model.

WUSN/site parameters are those that can be obtained directly from the deployment of the WUSN in the field. Because the signal transmitted from a buried node first travels through the soil, the power (PT (in dB)) at the soil surface depends on the moisture content in the soil. After traveling through the air with a free space pathloss proportional to 20 × log(D), where D is the hypotenuse distance of the buried sensor to the antenna, the signal is received at the base station antenna with a certain RSSI. Hence, one can approximate PT as being proportional to the sum of 20 × log(D) and RSSI. The distance (D) appears as a separate parameter in addition to the 20 × log(D) factor in the estimate of PT in order to capture propagation effects, such as multipath, which are not accounted for in the free space pathloss. These site parameters along with the measured weather and soil variables were used as input parameters for evaluating the machine learning algorithm.

Trial and error was used to select the optimum number of hidden layers and number of neurons. Table A1 in Appendix A shows the performance matrix of different combinations of hidden layers and number of neurons. Ultimately, a five-layer feed-forward neural network including three hidden layers, one input layer, and one output layer, with each hidden layer containing 55 neurons, was selected based upon performance in VWC predictions. The Levenberg–Marquardt algorithm was used for network training. To avoid overtraining, we used an early stopping training method. Model performance was assessed by comparing the coefficient of determination (R^2^), root mean square error (RMSE), and mean absolute error (MAE) of the estimated value of soil VWC to in situ, ground-truth measurements. All input and target parameters were normalized to a range between 0.2 to 0.8, as suggested by Cigizoglu (2003) [82]. Table 2 shows the performance of this model in training, validation, and testing with various combinations of input parameters. The six-input parameter model, containing all site and climate input parameters, predicted soil VWC with very good accuracy and low RMSE and MAE.

**Figure 7:**
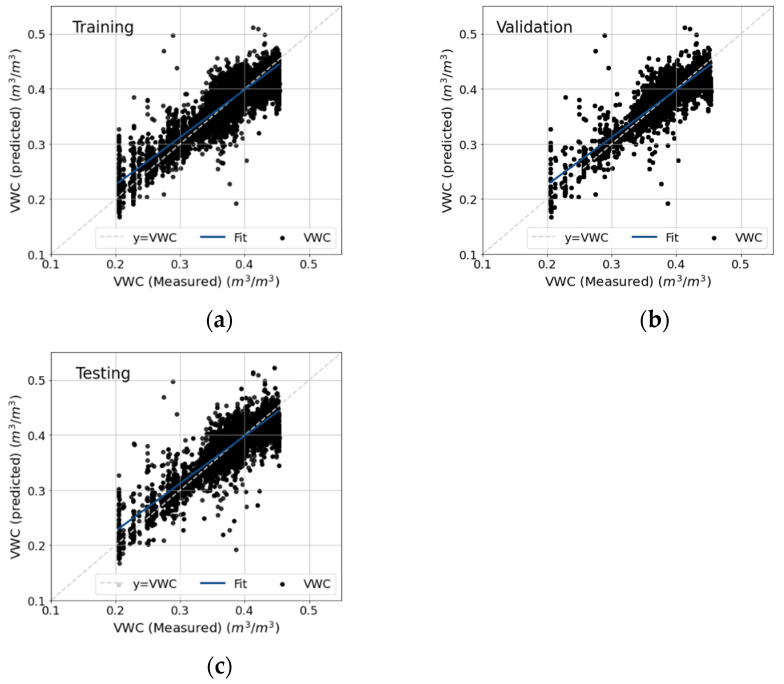
Measured and predicted soil volumetric water content (VWC) at training (**a**), validation (**b**), and testing (**c**) stages of the ANN–MLP model using input parameters: distance of the sensor node to the antenna (D), RSSI + 20 × log(D), relative humidity (RH), and soil and atmospheric temperature. Model is highlighted in bold on Table 2 as the best-performing model.

In contrast, running the model with only site input parameters, i.e., using only RSSI + 20 × log(D) and D as input parameters, substantially reduced the performance of the model, rendering unacceptable predictions (Table 2, two-parameter model). We determined that ST is an important variable in determining the model’s predictive ability, i.e., any model was substantially improved when ST was included as an input parameter (Table 2, three-, four-, and five-parameter models). Similarly, but to a lesser extent, the performance of the model could be further improved with the addition of AT and RH as input parameters (in addition to RSSI + 20 × log(D), D, and ST as inputs) leading to four- and five-parameter models (see Table 2). For example, Figure 7 shows measured and predicted soil VWC when using the ANN–MLP model with RSSI + 20 × log(D), D, ST, AT, and RH as input parameters. Including P in the model did not affect model performance (Table 2). Since P is infrequent, there were fewer inputs into the algorithm, which might have reduced its influence on model performance. Nevertheless, it is possible that the effect of P might already have been accounted for, indirectly, by RSSI and RH variations.

While RSSI is expected to and has been previously shown to be affected by soil VWC due to changes in the soil’s dielectric properties under limited testing conditions [10,36,38,69], our results summarized in Table 2 (and the seasonal examples highlighted in Figure 6) clearly show that RSSI alone is a poor consistent predictor of soil VWC, and we propose the use of this algorithm as an alternative method for estimating soil VWC with WUSNs. Indeed, dropping RH as an input parameter and using a four-parameter model also leads to good prediction of soil VWC (R^2^ = 0.82). The approach described above, while obviating the need for an expensive VWC sensor at every node, does require other soil and weather data. However, many algorithms developed to predict ST from AT can render this approach feasible [83,84,85,86].

We compared the performance of the six-parameter ANN–MLP model with two other commonly used machine learning algorithms: Support Vector Regression (SVR) [87,88,89] and Extreme Learning Machines (ELMs) [90,91]. We used these algorithms to predict soil moisture in the same way as for our ANN–MLP model, and the implementation details of the two models are described in Appendix B. The performance statistics of the two models are presented in Table 3, showing the best results across a collection of SVR kernel functions and ELM activation functions. Grid searches for these two models yielded poorer results than the ANN–MLP model. The best SVR model was found with a radial basis function (RBF) kernel which resulted in R^2^ = 0.56 and RMSE = 0.053 m^3^/m^3^ for the testing stage; the best ELM model was found with a sigmoid activation function, which resulted in R^2^ = 0.48 and RMSE = 0.06 m^3^/m^3^ for the testing stage. The reason for these models’ poor performance in comparison to the ANN–MLP model is unclear and beyond the scope of the current paper. We can conclude, however, that our deep learning approach surpasses the other surveyed methods even without a thorough grid search, and that the ANN–MLP model proves a useful tool in predicting geospatial VWC.

We now make a few comments regarding our analysis. Firstly, note that we did not include the polarization and antenna gain for the sensor node transmitters as input parameters. Polarization and antenna gains cannot be controlled precisely, especially in a real-world field deployment such as described in this paper. With ML, if measurements from each sensor are adequately represented in the training set, these, as well as other hidden factors, such as differences in terrain, will be learned by the model during the training phase. In the experiment, 92,000 datapoints were collected from 23 sensor nodes over 9 months, ensuring that measurements from each sensor node were well-represented in the training data. Furthermore, in estimating soil moisture, the ML model utilizes the relative difference in RSSI between wet soil and dry soil from each sensor, and hence, the absolute effect of polarization, antenna gain, and terrain do not need to be modeled accurately since these do not change with the level of moisture in the soil. Secondly, as noted in Section 3.2, some sites can have lower number of successful transmissions due to factors such as distance, topography, etc. To examine this effect, we tested the model with data from the sites that had >50% and <50% data packet transmission rates, and the difference in performance statistics during testing was not significant. However, this is a parameter that will likely need to be examined and tested in other site installations.

## 4. Conclusions

In this paper, we present the successful design and deployment of Thoreau 2.0, a scalable, low-power WUSN for subterranean sensing. The WUSN has been operating continuously in an agricultural field since September 2019, and we present data collected over 9 months (three seasons). High temporal and spatial resolutions were accomplished by measuring soil conditions at 30 min intervals and interpolating and mapping the sensor results over an approximated 530 m radius area. We showcase the ability of our WUSN to monitor and map real-time variations in soil VWC, ST, and EC. Our results show that such WUSNs can be reliably operated under “real-world” conditions and are scalable. Using the RSSI signal from the WUSN along with other inputs as proxies, we developed a deep learning model that can accurately predict and map an important parameter for soil ecology and agriculture: volumetric water content (or soil moisture). This enables soil moisture determination without the need for expensive sensors for direct VWC measurement.

## Figures and Tables

**Table 1:** Season descriptive parameter statistics.

	Fall-2019			Winter-2019			Spring-2020 **		
	Min	Avg	Max	Min	Avg	Max	Min	Avg	Max
VWC	0.27	0.38	0.45	0.34	0.39	0.45	0.33	0.39	0.45
EC	2.13	5.34	7.24	2.44	5.23	8.2	3.83	5.62	8.56
ST	0.3	8.7	26.0	0.0	2.4	5.9	1.5	12.2	23.9
AT	−15	4.7	29.5	−21.8	0.3	18.2	−4.7	10.7	31.9
RH	34.8	80.6	103.1	23.1	79.4	103.2	23.6	68.2	102.7
P	247			149			304		

VWC: volumetric water content (m^3^/m^3^), EC: soil electrical conductivity (dS/m), ST: soil temperature (°C), AT: air temperature (°C), RH: relative humidity (%), and P: cumulative precipitation (mm) ** Spring-2020 statistics for AT, RH, and cumulative precipitation are for 65 days.

**Table 2:** Results of ANN–MLP model with different input parameter combinations.

Input Parameters	Training			Validation			Testing		
	R^2^	RMSE (m^3^/m^3^)	MAE (m^3^/m^3^)	R^2^	RMSE (m^3^/m^3^)	MAE (m^3^/m^3^)	R^2^	RMSE (m^3^/m^3^)	MAE (m^3^/m^3^)
***Six-parameter model***									
RSSI + K, D, ST, AT, P, RH	0.917	0.01	0.006	0.812	0.013	0.007	0.809	0.012	0.007
***Two-parameter model***									
RSSI + K, D	0.249	0.026	0.016	0.259	0.026	0.016	0.237	0.026	0.016
***Three-parameter models***									
RSSI + K, D, ST	0.783	0.014	0.008	0.726	0.016	0.009	0.726	0.016	0.009
RSSI + K, D, AT	0.498	0.021	0.014	0.465	0.022	0.014	0.457	0.022	0.014
RSSI + K, D, P	0.262	0.026	0.016	0.248	0.026	0.016	0.249	0.025	0.016
RSSI + K, D, RH	0.314	0.025	0.015	0.262	0.026	0.016	0.254	0.026	0.016
***Four-parameter models***									
RSSI + K, D, ST, AT	0.821	0.013	0.008	0.764	0.015	0.009	0.742	0.015	0.009
RSSI + K, D, ST, RH	0.814	0.013	0.008	0.716	0.016	0.01	0.74	0.015	0.009
RSSI + K, D, ST, P	0.723	0.016	0.01	0.686	0.017	0.01	0.677	0.017	0.01
RSSI + K, D, AT, RH	0.65	0.018	0.012	0.593	0.019	0.013	0.536	0.02	0.013
RSSI + K, D, AT, P	0.483	0.022	0.014	0.47	0.022	0.014	0.431	0.022	0.015
RSSI + K, D, RH, P	0.306	0.025	0.016	0.254	0.026	0.016	0.257	0.025	0.016
ST, AT, P, RH	0.499	0.021	0.016	0.437	0.022	0.017	0.407	0.023	0.017
***Five-parameter models***									
RSSI + K, D, ST, AT, P	0.807	0.013	0.008	0.762	0.015	0.009	0.734	0.015	0.009
RSSI + K, D, ST, AT, RH *	**0.889**	**0.01**	**0.006**	**0.833**	**0.012**	**0.008**	**0.82**	**0.012**	**0.008**
RSSI + K, D, AT, RH, P	0.617	0.018	0.012	0.562	0.02	0.013	0.552	0.02	0.013
RSSI + K, D, ST, RH, P	0.745	0.015	0.01	0.697	0.016	0.01	0.697	0.016	0.01

D: Hypotenuse distance between sensor node and antenna, K: 20 × log(D), ST: soil temperature, AT: air temperature, P: precipitation, and RH: relative humidity. Stats: coefficient of determination (R^2^), root mean square error (RMSE), and mean absolute error (MAE). * Best overall model performance.

**Table 3:** Results of Support Vector Regression (SVR) and Extreme Learning Machines (ELMs) for training and testing stages.

	Kernel/Activation	Training			Testing		
		R^2^	RMSE (m^3^/m^3^)	MAE (m^3^/m^3^)	R^2^	RMSE (m^3^/m^3^)	MAE (m^3^/m^3^)
SVR	Linear	0.17	0.072	0.0544	0.17	0.073	0.055
	Sigmoid	0.17	0.072	0.055	0.18	0.073	0.054
	Poly	0.25	0.069	0.055	0.25	0.069	0.054
	RBF *	0.59	0.051	0.03	0.56	0.053	0.032
ELM	Sigmoid	0.47	0.058	0.0411	0.48	0.058	0.041
	Sine	0.44	0.06	0.044	0.43	0.06	0.044
	Tanh	0.42	0.061	0.045	0.39	0.062	0.045
	Triangular basis	0.43	0.06	0.043	0.43	0.061	0.044
	Hard limit	0.39	0.06	0.045	0.39	0.062	0.046
	Relu	0.39	0.045	0.06	0.38	0.045	0.063
	RBF *	0.43	0.044	0.06	0.41	0.045	0.062

* RBF: Radial Basis Function.

**Table A2:** Optimal training constants and kernel functions for developing SVM models.

Kernel Function	C	Epsilon	Gamma	Degree
Linear	0.1	0.1	-	-
Sigmoid	100	0.1	0.001	-
Poly	10	0.1	2	3
RBF	10	0.01	20	-

**Table A3:** Optimal hidden neurons for developing ELM model.

Activation Function	Number of Neurons in the Hidden Layer
Sigmoid	385
Sine	270
Tanh	180
Triangular basis	265
Hard limit	970
Relu	465
RBF	210

## Data Availability

Soil data were obtained from the open-access Thoreau soil database of Femi agricultural field and are available from Thoreau-Home (uchicago.edu) (accessed on 25 June 2020); the weather parameters obtained from the weather station installed at Femi agricultural field are available at https://www-esh.fnal.gov/pls/default/weather.html (accessed on 25 June 2020).

## Appendix A

Table A1 shows the performance statistics of different neural network architectures tested for volumetric water content (VWC) predictions.

**Table A1:** Performance statistics of different neural network architectures tested for volumetric water content (VWC) predictions.

S.No	Model Architecture	Training				Testing				Validation			
		R	R^2^	RMSE (m^3^/m^3^)	MAE (m^3^/m^3^)	R	R^2^	RMSE (m^3^/m^3^)	MAE	R	R^2^	RMSE (m^3^/m^3^)	MAE (m^3^/m^3^)
1	6-5-1	0.605	0.367	0.024	0.017	0.604	0.365	0.024	0.017	0.594	0.353	0.024	0.017
2	6-10-1	0.669	0.448	0.022	0.016	0.680	0.462	0.022	0.016	0.664	0.441	0.022	0.016
3	6-15-1	0.686	0.471	0.022	0.015	0.697	0.485	0.022	0.015	0.683	0.467	0.022	0.015
4	6-20-1	0.733	0.537	0.020	0.014	0.720	0.519	0.020	0.014	0.722	0.521	0.021	0.015
5	6-25-1	0.737	0.543	0.020	0.014	0.723	0.522	0.020	0.014	0.730	0.532	0.020	0.014
6	6-30-1	0.739	0.546	0.020	0.014	0.738	0.545	0.020	0.014	0.746	0.556	0.020	0.014
7	6-35-1	0.732	0.535	0.020	0.014	0.714	0.510	0.021	0.014	0.714	0.508	0.020	0.014
8	6-40-1	0.756	0.572	0.019	0.014	0.758	0.575	0.019	0.014	0.758	0.574	0.020	0.014
9	6-45-1	0.789	0.622	0.018	0.013	0.777	0.603	0.019	0.013	0.774	0.599	0.019	0.013
10	6-50-1	0.792	0.628	0.018	0.013	0.781	0.610	0.019	0.013	0.784	0.614	0.018	0.013
11	6-55-1	0.786	0.618	0.018	0.013	0.776	0.603	0.019	0.013	0.773	0.598	0.019	0.013
12	6-60-1	0.794	0.630	0.018	0.013	0.785	0.617	0.019	0.013	0.781	0.610	0.019	0.013
13	6-65-1	0.789	0.623	0.018	0.013	0.781	0.610	0.018	0.013	0.784	0.614	0.018	0.013
14	6-70-1	0.802	0.643	0.018	0.013	0.787	0.619	0.018	0.013	0.769	0.590	0.019	0.013
21	6-5-5-1	0.613	0.376	0.024	0.017	0.611	0.374	0.023	0.016	0.608	0.370	0.024	0.017
22	6-10-10-1	0.766	0.586	0.019	0.014	0.752	0.566	0.019	0.014	0.771	0.594	0.019	0.013
23	6-15-15-1	0.817	0.668	0.017	0.012	0.809	0.654	0.018	0.012	0.805	0.648	0.017	0.012
24	6-20-20-1	0.841	0.708	0.016	0.011	0.825	0.681	0.017	0.011	0.835	0.698	0.017	0.011
25	6-25-25-1	0.867	0.751	0.015	0.010	0.848	0.718	0.016	0.011	0.852	0.726	0.015	0.010
26	6-30-30-1	0.875	0.765	0.014	0.010	0.859	0.737	0.015	0.010	0.870	0.757	0.015	0.010
27	6-35-35-1	0.888	0.788	0.014	0.009	0.880	0.775	0.014	0.010	0.877	0.768	0.014	0.010
28	6-40-40-1	0.857	0.734	0.015	0.010	0.843	0.710	0.016	0.011	0.837	0.699	0.016	0.011
29	6-45-45-1	0.909	0.827	0.012	0.008	0.882	0.778	0.014	0.009	0.891	0.794	0.014	0.009
30	6-50-50-1	0.887	0.786	0.014	0.009	0.861	0.740	0.015	0.010	0.863	0.744	0.015	0.010
31	6-55-55-1	0.883	0.780	0.014	0.009	0.847	0.714	0.016	0.010	0.849	0.718	0.016	0.010
32	6-60-60-1	0.904	0.817	0.013	0.008	0.871	0.758	0.015	0.009	0.872	0.758	0.015	0.009
33	6-65-65-1	0.934	0.872	0.011	0.007	0.886	0.779	0.014	0.008	0.892	0.794	0.013	0.008
34	6-70-70-1	0.916	0.838	0.012	0.008	0.872	0.758	0.015	0.009	0.875	0.762	0.014	0.009
35	6-5-5-5-1	0.718	0.516	0.021	0.015	0.707	0.499	0.021	0.015	0.707	0.499	0.021	0.015
36	6-10-10-10-1	0.828	0.686	0.017	0.011	0.817	0.667	0.017	0.012	0.822	0.676	0.017	0.012
37	6-15-15-15-1	0.850	0.723	0.016	0.011	0.826	0.682	0.017	0.011	0.835	0.697	0.016	0.011
38	6-20-20-20-1	0.883	0.781	0.014	0.009	0.867	0.752	0.015	0.010	0.867	0.751	0.015	0.010
39	6-25-25-25-1	0.898	0.806	0.013	0.009	0.875	0.765	0.014	0.009	0.881	0.776	0.014	0.009
40	6-30-30-30-1	0.910	0.828	0.012	0.008	0.883	0.780	0.014	0.009	0.891	0.794	0.014	0.009
41	6-35-35-35-1	0.908	0.825	0.012	0.008	0.879	0.770	0.014	0.009	0.885	0.782	0.014	0.009
42	6-40-40-40-1	0.917	0.840	0.012	0.008	0.890	0.791	0.014	0.009	0.883	0.778	0.014	0.009
43	6-45-45-45-1	0.934	0.872	0.011	0.007	0.898	0.805	0.013	0.008	0.897	0.803	0.013	0.008
44	6-50-50-50-1	0.918	0.842	0.012	0.008	0.883	0.778	0.014	0.009	0.896	0.802	0.013	0.009
45	6-55-55-55-1	0.958	0.917	0.01	0.006	0.904	0.812	0.013	0.007	0.899	0.809	0.012	0.007
46	6-60-60-601	0.926	0.858	0.011	0.007	0.884	0.777	0.014	0.009	0.893	0.795	0.014	0.008
47	6-65-65-65-1	0.916	0.839	0.012	0.008	0.887	0.786	0.014	0.009	0.886	0.784	0.014	0.009
48	6-70-70-70-1	0.915	0.838	0.012	0.008	0.882	0.776	0.014	0.009	0.882	0.776	0.014	0.009

## Appendix B

We implemented our SVR models using the out-off-the-box SVR function in Python’s scikit-learn package [88,90]. The SVR function in Python’s scikit-learn package implements epsilon-supported SVR wherein no penalty is associated with training loss within a certain distance ε from the target data. A randomized grid search was used for hyperparameter tuning 89, using scikit-learn’s GridSearchCV function. The search is defined for the regularization parameter C = {0.001, 0.1, 1, 10, 100, 500, 1000, 2500}; the epsilon-support region size ε = {0.00001, 0.0001, 0.001, 0.005, 0.01, 0.1, 1}; and the kernel coefficient γ = {0.0001, 0.001, 0.01, 0.1, 1, 2, 10, 20}; γ was also allowed the values ‘auto’ and ‘scale’ according to scikit-learn’s implementation, which, respectively, use the inverse of the number of input features and the inverse of the number of input features times the variance of the input data. Table A2 shows the optimal C, ε, γ, and degree values for the respective kernel functions for VWC prediction.

For soil moisture prediction, the associated ELM neural network involved an input layer with six neurons (one per input parameter), one hidden layer, and one output layer with one neuron. MATLAB was used to develop an ELM model with a specified number of hidden neurons with a specified activation function (tested sigmoid, sine, tanh, triangular basis, hard limit, ReLu, and RBFs). The number of neurons between 5 and 1000 with an increment of five were tested in the hidden layer of the ELM models. Table A3 shows the optimal hidden neurons for different activation functions.
